# Sitagliptin and Simvastatin Interaction Causing Rhabdomyolysis and AKI

**DOI:** 10.1155/2019/2601537

**Published:** 2019-02-26

**Authors:** Chinmay Patel, Courtney Thompson, Megan Copley-Harris, Yousef Hattab

**Affiliations:** ^1^Clinical Professor of Nephrology, University of Pikeville-Kentucky College of Osteopathic Medicine, Division of Nephrology, Pikeville Medical Center, Pikeville, KY, USA; ^2^Resident, Family Practice Residency Program, Pikeville Medical Center, Pikeville, KY, USA; ^3^Department of Hospital Medicine, Pikeville Medical Center, Pikeville, KY, USA; ^4^Division of Pulmonary and Critical Care, Pikeville Medical Center, Pikeville, KY, USA

## Abstract

We report a case of rhabdomyolysis and severe acute kidney injury (AKI) requiring dialysis in a 69-year-old male who was recently started on sitagliptin while on chronic simvastatin therapy. This potential interaction is not included in the package insert for sitagliptin. A comprehensive literature review revealed six previous reports of rhabdomyolysis due to drug interaction between sitagliptin and statins including simvastatin, lovastatin, and atorvastatin. Of these six cases, only two had developed rhabdomyolysis-associated AKI, none of which were severe enough to require dialysis. As patients are commonly prescribed statins and sitagliptin for treatment of dyslipidemia and diabetes, health care professionals should be aware of this potential drug interaction and closely monitor their patients for signs and symptoms of rhabdomyolysis and AKI. This case highlights the importance of conducting further studies on the risk of muscular toxicity of sitagliptin especially when administered concurrently with statins.

## 1. Introduction

Sitagliptin belongs to a class of oral antihyperglycemic drugs known as dipeptidyl peptidase-4 (DPP-4) inhibitors [1]. Statin use for management of dyslipidemia is common in patients with diabetes. We present a patient on long-term simvastatin therapy who developed severe rhabdomyolysis and AKI soon after starting treatment with sitagliptin.

## 2. Case Presentation

A 69-year-old white male with history of hypertension, noninsulin-dependent diabetes, chronic kidney disease stage 3, hyperlipidemia, coronary artery disease, and congestive heart failure was brought to hospital with complaints of generalized malaise, muscle weakness with pain in both his lower extremities, and inability to ambulate for last 4-5 days. He had developed these symptoms within 2 days of starting sitagliptin (Januvia 100 mg) therapy. His other medications included simvastatin, amlodipine, carvedilol, clopidogrel, gabapentin, glipizide, hydrochlorothiazide, metformin, quinapril, and ezetimibe. He gave history of similar weakness when he was started on sitagliptin therapy few months ago, so he discontinued it by himself. His primary care physician however restarted it again at recent follow-up, when the patient refused insulin therapy.

In the emergency department, the patient was hypotensive with a blood pressure of 90/60 mm Hg with a pulse of 70 bpm. On examination, he had marked proximal muscle weakness of bilateral lower extremities. There was mild tenderness to palpation of both thighs. The rest of physical examination was unremarkable. Laboratory data revealed serum creatinine of 9.1 mg/dL with blood urea nitrogen of 130 mg/dL. 3 months prior to presentation, the patient's serum creatinine was 1.2 mg/dL with urinalysis showing 1+ proteinuria and an estimated glomerular filtration rate of 62 ml/min/1.73 m^2^. Creatine kinase (CK) level came back significantly elevated at 43,900 U/L. Potential causes of rhabdomyolysis like trauma, seizures, hypophosphatemia, drug and alcohol abuse, hypothyroidism, and vitamin D deficiency were ruled out. He had been on a stable dose of simvastatin 80 mg daily for more than 10 years. Intravenous hydration with bicarbonate therapy was initiated; however, the patient was noted to be oliguric and required initiation of hemodialysis therapy. AKI was presumed to be due to rhabdomyolysis-induced acute tubular necrosis and so kidney biopsy was not performed. Both sitagliptin and simvastatin were promptly discontinued. Rhabdomyolysis gradually improved; however, our patient remained oliguric and dialysis-dependent at the time of discharge at 2 weeks. The patient recovered his kidney function and was taken off hemodialysis after 2 months following his discharge.

## 3. Discussion

Simvastatin belongs to the family of 2-hydroxy-3 methylglutaryl coenzyme A (HMG-CoA) reductase inhibitors used for treatment of dyslipidemia. It is metabolized by hepatic cytochrome P450 (CYP3A4) enzymes and thus has significant drug interactions with other CYP3A4 inhibitors [2].

Sitagliptin belongs to a class of oral antihyperglycemic drugs known as dipeptidyl peptidase-4 (DPP-4) inhibitors, which act by inhibiting the inactivation of incretin hormones, namely, glucagon-like peptide-1 (GLP-1) and glucose-dependent insulinotropic polypeptide (GIP) by the DPP-4 enzyme. Increased levels of incretin hormones stimulate the glucose-dependent synthesis and release of insulin from the pancreatic *β* cells. GLP-1 also suppresses hepatic gluconeogenesis by inhibiting the pancreatic *α* cells. Sitagliptin is primarily excreted via renal tubular secretion and requires dose adjustment in patients with chronic kidney disease [1]. Our patient had normal serum creatinine when he was started on 100 mg sitagliptin therapy. His previous urinalysis did however show 1+ proteinuria suggestive of mild degree of chronic kidney disease. About 16% of the drug is metabolized by the CYP3A4 and CYP2C8 enzymes. Side effects commonly seen with sitagliptin include headache, nasopharyngitis, diarrhea, nausea, and increased serum creatinine [1]. Rhabdomyolysis is a potentially life-threatening clinical condition that involves breakdown of muscle tissue with a resultant release of myoglobin and other cellular contents into the systemic circulation. The major causes of rhabdomyolysis include trauma or crush injuries to the skeletal muscles, hyperthermia, electrolyte imbalance, seizures, genetic muscle diseases, and sometimes as a result of drug interactions [3].

Our patient developed his symptoms soon after sitagliptin was added to his medical therapy. He had tolerated simvastatin therapy well for many years with no recent change in the dose, suggesting that rhabdomyolysis and AKI was precipitated by initiation of sitagliptin therapy. The sitagliptin package insert does not mention any drug interaction with statins [1]. A comprehensive literature review revealed six previous reports of rhabdomyolysis due to drug interaction between sitagliptin and statins including simvastatin, lovastatin, and atorvastatin [4–9] (Table 1). Of these, only two cases had rhabdomyolysis-associated AKI neither of which were severe enough to require dialysis [4, 6]. The mechanisms proposed for this drug interaction include nephrotoxicity of sitagliptin causing reduced renal excretion of simvastatin and subsequently dose-related muscle breakdown. Other mechanisms suggest an interaction of statin and sitagliptin at the level of hepatic CYP3A4 as both are metabolized by the same enzyme and can potentially compete for it resulting in increased serum statin levels precipitating statin-induced rhabdomyolysis [4, 5]. Only three of the reported seven cases so far had developed AKI, thus supporting the latter theory of drug interaction at the hepatic CYP3A4 level. A recent report however found no evidence of an interaction between DPP-4 inhibitors and statins and showed that DPP-4 inhibitors themselves carry an increased risk of myopathy [10].

## 4. Conclusion

In summary, we report a patient who developed acute onset rhabdomyolysis and severe AKI after sitagliptin was added to his chronic simvastatin therapy. We believe that the interaction between these two drugs caused the rhabdomyolysis. Clinicians should be aware of this potential drug interaction and closely monitor their patients for signs and symptoms of rhabdomyolysis and AKI. This case highlights the importance of conducting further studies on the risk of muscular toxicity of sitagliptin especially when administered concurrently with other statins.

## Figures and Tables

**Table 1:** Cases of rhabdomyolysis precipitated by sitagliptin-statin drug interaction.

Reference	Medications	Duration of statin therapy	Duration of sitagliptin therapy	Peak CK level (U/L)	Associated AKI
[4]	Atorvastatin 20 mg + sitagliptin 100 mg	1 year	3 weeks	49,875	Yes
[5]	Atorvastatin 40 mg + sitagliptin 100 mg	Several years	1 week	13,456	No
[6]	Simvastatin 80 mg + sitagliptin 100 mg	4 months	3 weeks	22,000	Yes
[7]	Lovastatin 40 mg + sitagliptin 100 mg	12 years	19 days	3770	No
[8]	Atorvastatin + sitagliptin	5 years	6 months	109,710	No
[9]	Atorvastatin 80 mg + sitagliptin	N/A	N/A	1253	No
Our case	Simvastatin 80 mg + sitagliptin 100 mg	>10 years	5 days	43,900	Yes

CK: creatine kinase; AKI: acute kidney injury; N/A: not available.
